# Return-to-Sport Rates After Hip Arthroscopy for Femoroacetabular Impingement Syndrome in Flexibility Sports Athletes: A Systematic Review

**DOI:** 10.1177/19417381231217503

**Published:** 2023-12-28

**Authors:** Muyiwa Ifabiyi, Milin Patel, Dan Cohen, Nicole Simunovic, Olufemi R. Ayeni

**Affiliations:** † Division of Orthopaedic Surgery, Department of Surgery, McMaster University, Hamilton, Ontario, Canada; ‡ Faculty of Medicine, Michigan State University, Michigan; § Department of Health Research Methods, Evidence, and Impact, McMaster University, Hamilton, Ontario, Canada; ‖ Faculty of Health Sciences, McMaster University, Hamilton, Canada

**Keywords:** femoroacetabular impingement, flexibility sports, hip arthroscopy, outcomes

## Abstract

**Context:**

Femoroacetabular impingement syndrome (FAIS) is a common cause of hip pain in young adults. Flexibility athletes represent an interesting subset due to the extreme range of motion requirements of their sport.

**Objective:**

The objective of this review was to provide a summary of the outcomes of hip arthroscopy for FAIS in patients who participate in flexibility sports.

**Data Sources:**

Three online databases (Medline, Embase, and PubMed) were searched from database inception (1946, 1974, and 1966, respectively) to January 10, 2023.

**Study Selection:**

Studies were screened for literature addressing surgical outcomes for flexibility athletes undergoing hip arthroscopy for FAIS.

**Study Design:**

Systematic review.

**Level of Evidence:**

Level 4.

**Data Extraction:**

Various patient-reported outcomes that evaluated the efficacy of hip arthroscopy in this patient population were abstracted and presented in descriptive and analytical format. Abstraction was performed by 2 reviewers.

**Results:**

Overall, a total of 8 Level 3 or 4 studies and 295 patients (312 hips) were included in this review. The pooled standardized mean differences for the Visual Analog Scale for pain score, Modified Harris Hip Score, Hip Outcome Score - Activity of Daily Living scale, and Hip Outcome Score - Sport-Specific Subscale all demonstrated significant improvement after undergoing arthroscopy for FAIS between 12 and 116 months (N = 175, -1.97, 95% CI -2.5 to -1.4, *P* < 0.01, *I*^2^ = 76%; N = 211, 1.82, 95% CI 1.49 to 2.16, *P* < 0.01, *I*^2^ = 52%; N = 164, 1.75, 95% CI 1.42 to 2.05, *P* < 0.01, *I*^2^ = 28%; N = 211, 1.71, 95% CI 1.38 to 2.04, *P* < 0.01, *I*^2^ = 52%, respectively). Across 289 patients, 75.6% to 98% returned to sport at a similar or higher level than presurgery.

**Conclusion:**

This review demonstrates a trend of improvement in patient-reported pain, function, quality of life, and return to sport at a minimum of 12 months among flexibility athletes after hip arthroscopy to treat FAIS.

Femoroacetabular impingement syndrome (FAIS) is a recognized condition noted to be a common cause of hip pain in the young adult. This condition may be a precursor to the development of degenerative conditions in the hip.^6^ Hip arthroscopy has been shown to be an effective treatment option for correcting FAIS for symptom relief.^2,9,18^

Athletes represent an interesting subset of the generalized population that encounter frequent occurrences of FAIS.^1^ Athletes can be further categorized into multiple sport types, including cutting, contact, asymmetric/overhead, endurance, and flexibility.^15^ A compelling population that can sustain injuries associated with FAIS are engaged in flexibility sports such as dance, gymnastics, yoga, cheerleading, figure skating, and martial arts. Considering that athletes in flexibility sports require a greater range of motion (ROM) in multiple planes, understanding outcomes of FAIS treatment in this population is of relevance.

Current knowledge has shown that surgical management of FAIS demonstrates superior results to that of nonsurgical approaches. However, there is limited information regarding the benefits of arthroscopy specifically in the flexibility athlete population.^4^

The purpose of this systematic review was to investigate and evaluate the outcomes of hip arthroscopy in the treatment of FAIS in flexibility athletes. The study hypothesis was that hip arthroscopy offers favorable outcomes demonstrated by various patient-reported outcome measures.

## Methods

This systematic review was conducted in accordance with the PRISMA (Preferred Reporting Items for Systematic Reviews and Meta-Analyses) guidelines for conducting and reporting systematic reviews.^14^

### Search Strategy

Three online databases (Medline, Embase, and PubMed) were searched from database inception (1946, 1974, 1966, respectively) to January 10, 2023, for literature addressing surgical outcomes for flexibility athletes undergoing hip arthroscopy for FAIS. Broad search terms used to identify all eligible studies included “Hip,” “arthroscopy,” and “Femoroacetabular impingement” (Appendix Table A1, available in the online version of this article).

### Study Screening

Studies identified during the comprehensive search were screened for the combined title and abstract, and full-text stages by 2 reviewers independently and in duplicate. Disagreements during the title and abstract screening stage were carried forward to the next stage for more in-depth review. Any disagreements at the full-text stage were resolved by consensus between the reviewers, and a senior author was consulted for any remaining discrepancies. The references of the included studies then underwent manual screening to identify any additional articles.

### Assessment of Study Eligibility

The research question and study eligibility were established a priori. Inclusion criteria were as follows: (1) therapeutic studies of all levels of evidence; (2) English language studies; (3) human studies; and (4) studies reporting the precise outcomes for athletes in flexibility sports undergoing primary arthroscopic hip surgery for FAIS. Flexibility sports included, but were not limited to, sports such as dance, gymnastics, yoga, cheerleading, figure skating, and martial arts. Exclusion criteria were as follows: (1) cadaveric studies; (2) conference abstracts; (3) review papers; (4) technique guides; (5) case series of <5 patients; (6) textbook chapters; (7) patients undergoing hip arthroscopy for pathology other than FAIS; and (8) patients not participating in flexibility related sports. Moreover, the methods section of all studies was reviewed in detail and when 2 studies reported on an overlapping group of patients by the same senior author, the study with either the most relevance to our study or most recent follow-up was included.

### Assessment of Agreement

Cohen’s kappa (κ) statistic was used to evaluate inter-reviewer agreement at all screening and quality assessment stages. Agreement was classified a priori as follows: κ of 0.81 to 0.99 was considered near-perfect agreement, κ of 0.61 to 0.80 was substantial agreement, κ of 0.41 to 0.60 was moderate agreement, κ of 0.21 to 0.40 was fair agreement, 0.21 to 0.40 slight agreement, and a κ value of ≤0.20 was considered poor agreement.^7^

### Quality Assessment

The methodological quality of nonrandomized studies was evaluated using the Methodological Index for Nonrandomized Studies (MINORS) criteria.^16^ Using the items on the MINORS checklist, noncomparative studies can achieve a maximum score of 16, while comparative studies can achieve a maximum score of 24. Noncomparative studies were categorized a priori based on a previous systematic review by our group as follows: scores 0 to 4 indicate very low-quality evidence, scores 5 to 7 indicate low quality, scores 8 to 12 indicate fair quality, and scores ≥13 indicate high quality. For comparative studies including randomized controlled trials (RCTs), categorization was as follows: 0 to 6; very low quality; 7 to 10, low quality; 11 to 15, fair quality; 16 to 20, good quality; and ≥20, high quality.

### Data Abstraction

Two reviewers independently abstracted relevant data from included articles and recorded data into Microsoft Excel spreadsheets created a priori (Version 2007, Microsoft). Demographic data of the patient population were recorded, including information on patient age, follow-up, and return-to-sport rates. Data on various patient-reported outcomes were abstracted. Furthermore, studies reporting on >1 group eligible for study inclusion were presented as different studies for data abstraction purposes.

### Statistical Analysis

The standardized mean difference for continuous, patient-reported outcomes was pooled using a random effects model and displayed using forest plots. For other outcomes that were reported less uniformly, or indicated high heterogeneity that would preclude pooling the data, results were presented descriptively. Descriptive results include means and standard deviations, ranges, and percentages.

## Results

### Study Characteristics and Quality

**Figure 1.**
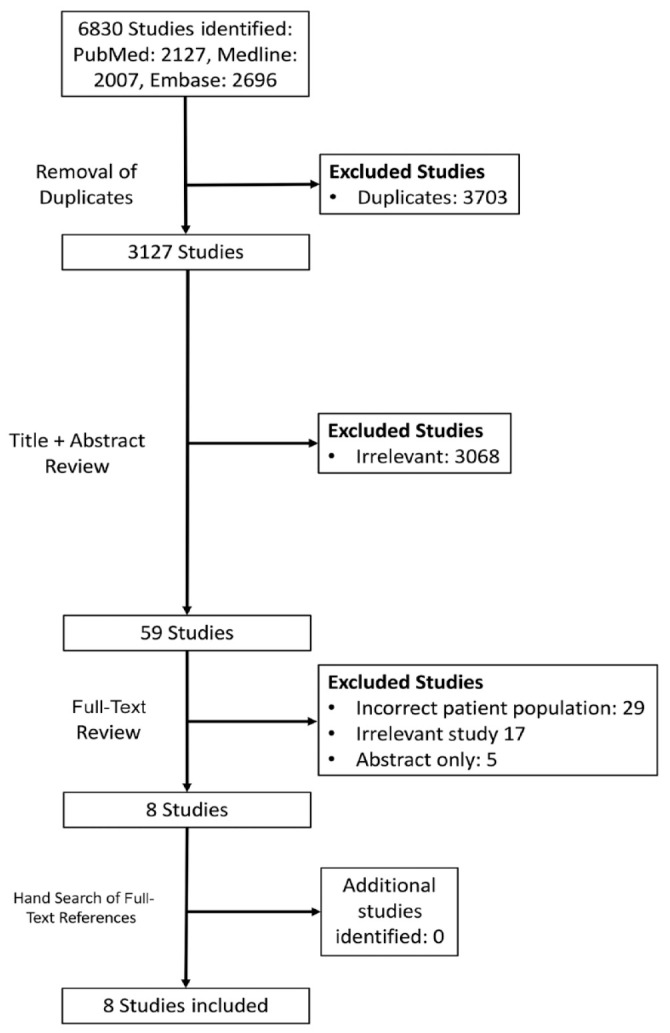
PRISMA flow diagram. PRISMA. Preferred Reporting Items for Systematic Reviews and Meta-Analyses.

The initial search yielded 6830 studies, which, after duplicate removal, was reduced to 3127. Systematic screening and assessment of eligibility resulted in 8 full-text studies that satisfied the inclusion criteria (Figure 1).

Overall, a total of 295 patients underwent surgery for FAIS, which encompassed 312 hips (Table 1). Studies were published between 2017 and 2022. The mean number of patients per included study was 49.13 (range, 25-66). Across 7 studies that reported demographic data, the mean age was 28.5 years and 81% were women. All studies had a minimum follow-up time of either 12 months or 24 months, with mean follow-up times ranging from 23 to 37.5 months (minimum to maximum range, 12-116 months).

**Table 1.** Study characteristics and rehabilitation protocol

Author (Year)	Study Design (Level of Evidence)	Number of Patients/Hips	Follow-up Time (Range), Months	Postop Rehab	MINORS
Saks (2022)	Cohort study (3)	47/50 (flexibility sports)	37.5 ± 10.4	Minimum 3 months starting day 1	17/24
Keating (2020)	Retrospective analysis (4)	25 (22 completed follow-up)	24 minimum	4-Phase rehab protocol lasting an average of 16 to 18 weeks	11/16
Mas Martinez (2020)	Case series (4)	36 (flexibility sports)	24 minimum	Postop rehab protocols included progressive activities for a period of up to 16 weeks. Return to sports allowed if patients demonstrate the ability to perform pain-free running, jumping, lateral agility drills, and single-leg squats	16/24
Larson (2020)	Case series (4)	63 dancers (77 hips)	36 (range, 12-116)	Patients were typically kept toe touch weightbearing postop after arthroscopy for 2 to 3 weeks without a brace or continuous passive motion machine. Physical therapy including early mobilization and ROM with circumduction or well-leg cycling was begun postoperative day 0 or 1. Return-to-dance-specific activities began as early as 3 months with full return to dance when pain-free ROM and normal functional progression were achieved at the discretion of a dance-focused physical therapist	9/16
Ukwuani (2019)	Case series (4)	64	23.0 ± 12.2 (12-60)	Patients went through the same 4-phase rehab protocol lasting 24 to 32 weeks	10/16
Frank (2018)	Retrospective analysis (4)	42/45	12 minimum	4-Phase rehab protocol lasting an average of 32 weeks	11/16
Weber (2017)	Retrospective comparative (3)	15 Flexibility	30.2 ± 4.8	Patients were kept on crutches for 3 weeks with a 9-kilogram, flat-foot weightbearing restriction. Passive and gentle active motion, including circumduction, was encouraged to prevent stiffness. At 3 weeks, patients progressed to weightbearing as tolerated without crutches or a brace. Also at 3 weeks, athletes progressed to hip ROM exercises including gentle stretching in all planes and progressed core and hip muscle strengthening. At 6 weeks, closed chain exercises began and stretching was advanced. At 12 weeks, patients participated in running using an antigravity treadmill and performed plyometrics. Patients were typically cleared to return to sports at 4 to 6 months after surgery based on their individual milestones in therapy	10/16
Mohan (2017)	Therapeutic case series (4)	3 Flexibility	34 (24-77)	5-Phase rehab	13/16

MINORS, methodological index for nonrandomized studies; postop, postoperative; rehab, rehabilitation; ROM, range of motion.

Among the 8 studies included in this review, the 6 Level 4 evidence studies included 2 retrospective analyses and 4 case series. The 2 Level 3 evidence studies included 2 retrospective cohort studies (Table 1). The mean MINORS score was 10.83 (range, 9-13) for noncomparative studies, indicating fair quality. The mean MINORS score was 17.0 (range, 16-18) for comparative studies indicating good quality (Table 1). Agreement between the reviewers was nearly perfect at the title/abstract (κ = 0.84) and full-text (κ = 0.85) stages.

### FAIS Morphology Management Details

The mean pre- and postoperative alpha angles in 5 studies were 58 (SD 3.0) and 41.5 (SD 5.2), respectively.^3,5,8,11,15^ The mean pre- and postoperative lateral center edge and tonnis angles were was 38.33 (SD 15.1), 34.01 (SD 8.3), 5.7 (SD 2.1), and 4.9, respectively.^3,5,8,11,15^ In all, 217 and 124 patients underwent surgery for cam and pincer deformities, respectively, and 254 patients underwent repair for labral pathology (Table 2).^3,5,8,15,19^

**Table 2.** Radiographic parameters

Author (Year)	No. of Patients/Hips	Female, %	Mean age (range), years	Preoperative Alpha Angle	Postoperative Alpha Angle	Preoperative Lateral Center Edge Angle	Postoperative Lateral Center Edge Angle	Type of FAI (Cam, Pincer, mixed)	Concurrent Pathology (Labral Tear, Ligamentum Teres Tear)
Saks (2022)	47/50 (flexibility sports)	96	19.5 ± 7.3	53.1 ± 9.6	43.3 ± 6.1	28.9 ± 5.1	NR	NR	Labral treatments (selective debridement 11 (22%), repair 38 (76%), reconstruction 1 (2%)
Keating (2020)	25 (22 completed follow-up)	100	38.1 ± 10.8	57.9 ± 7.3	36.1 ± 4.1	32.1 ± 4.6	30.9 ± 5.2	All 25 Cam (100%)	22 (100%) labral tears
Mas Martinez (2020)	36 (flexibility sports)	26.5 (49/185)	36.7 (range, 18-50)	58.6±8.6		32.6 ± 8.3	NR	17 Cam (47.2%) 5 Pincer (14%) 14 Mixed (38.8%)	NR
Larson (2020)	63 dancers (77 hips)	61/63	21.2 (range, 13-62)	61.3 (range, 40-90)	47.9 (range, 38-85)	65.2 (range, 45-84)	43.4 (range, 35-83)	73 Cam (94.8%) 25 (32.5%) pincer	73 (95%) required labral repairs
Ukwuani (2019)	64	97	22.3 ± 9.4	NR	NR	NR	NR	Radiographic: Cam 63 (98%) Pincer 6 (9%) Intraoperatively: Cam 59 (92%) Pincer 54 (84%)	All 64 patients had evidence of a labral tear intraoperatively; 62 patients underwent labral repair, whereas 2 underwent selective labral debridement
Frank (2018)	42/45	90	35 ± 9	59.20 ± 15.26	38.79 ± 9.9	32.87 ± 9.17	27.74 ± 7.9	43 Cam (96%) 40 pincer (89%) 38 mixed (84%)	45 (100%) labral tear, 11 (24%) cartilage delamination
Weber (2017)	15 (flexibility sports)	60	26.8 ± 7.8	NR	NR	NR	NR	NR	NR
Mohan (2017)	3 (flexibility sports)	NR	NR	NR	NR	NR	NR	NR	NR

FAI, femoroacetabular impingement; NR, not reported.

### Capsular Management Details

The capsulotomy technique was reported by all studies. Of the 8 studies covering 113/295 patients, 3 reported performing interportal capsulotomy and, of the 8 studies covering 182/295 patients, 5 reported performing T-shaped capsulotomy. All surgeries were performed arthroscopically. In 6 of the 8 studies covering 242 patients, the capsule was repaired. Of the 8 studies, 2 reported capsulotomy management changing during their inclusion period and therefore did not report routine repair in all hips. Saks et al^15^ reported a 96% repair rate, whereas Mohan et al^13^ reported a 56% repair rate.

### Rehabilitation Protocol

All studies reported on their postoperative rehabilitation protocol (Table 1). The most common was a 4-phase rehabilitation protocol, encompassing joint protection, gait maintenance, single leg squats and strengthening, and sports participation. Only 4 of the 8 studies reported on bracing practices. Saks et al,^15^ which included 47 patients and 50 hips, was the only study that placed patients in a hinged hip brace postoperatively. All studies reported rehabilitation protocols that started day 1 postoperatively, with most studies reporting the initial use of crutches for 2 to 4 weeks while performing passive ROM exercises (Table 1).

### Patient-Reported Outcomes

**Figure 2.**
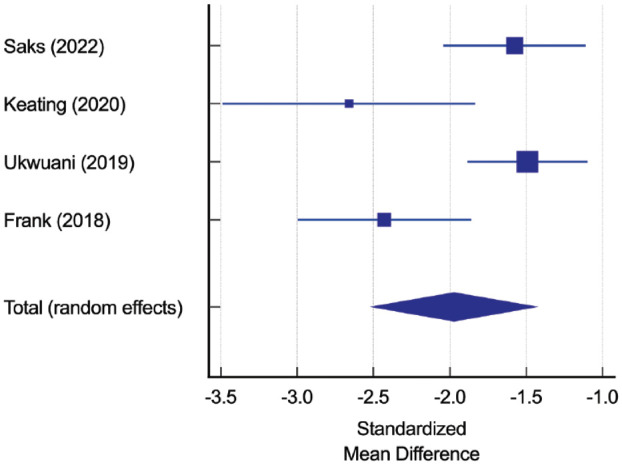
Standardized mean difference for VAS score. VAS, Visual Analog Scale.

The standardized mean difference for the Visual Analog Scale (VAS) for pain score in 4 studies comprising 175 patients was -1.97 (95% CI -2.5 to -1.4, *P* < 0.01, *I*^2^ = 76%) (Figure 2), indicating significantly improved pain at a minimum of 12 months’ follow-up and a range of 12 to 60 months.^3,5,15,19^

**Figure 3.**
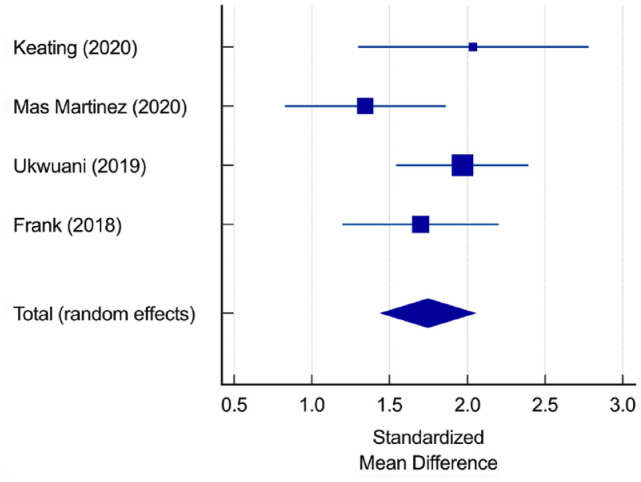
Standardized mean difference for mHHS. mHHS, modified Harris Hip Score.

The standardized mean difference for the Modified Harris Hip Score (mHHS) in 5 studies comprising 211 patients was 1.82 (95% CI 1.49 to 2.16, *P* < 0.01, *I*^2^ = 52%) (Figure 3), indicating a significant improvement at a minimum of 12 months and a range of 12 to 60 months.^3,5,11,15,19^

**Figure 4.**
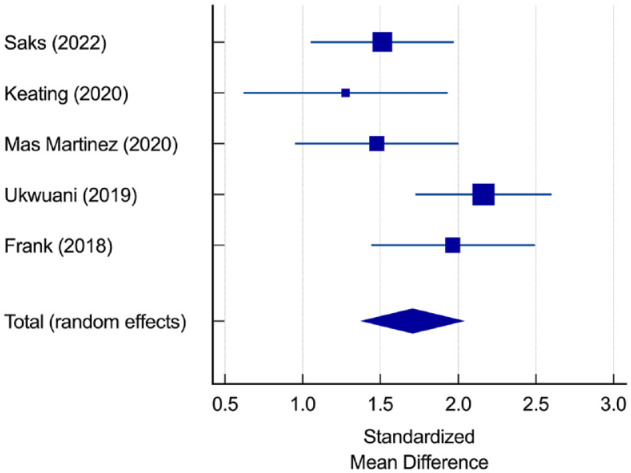
Standardized mean difference for HOS-ADL score. HOS-ADL, Hip Outcome Score - Activity of Daily Living.

**Figure 5.**
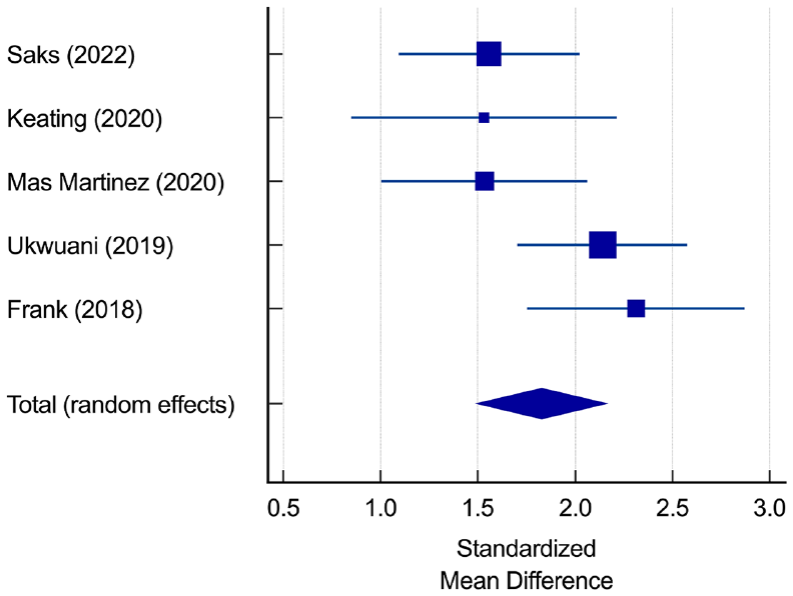
Standardized mean difference for HOS-SSS score. HOS-SSS, Hip Outcome Score - Sport Specific Subscale.

The standardized mean difference for the Hip Outcome Score - Activity of Daily Living (HOS-ADL) scale in 4 studies comprising 164 patients was 1.75 (95% CI 1.42 to 2.05, *P* < 0.01, *I*^2^ = 28%) (Figure 4),^3,5,11,19^ and the standardized mean difference for the Hip Outcome Score - Sport-Specific Subscale (HOS-SSS) in 5 studies comprising 211 patients was 1.71 (95% CI 1.38 to 2.04, *P* < 0.01, *I*^2^ = 52%) (Figure 5), indicating a significant improvement at a minimum of 12 months and a range of 12 to 60 months.^3,5,11,15,19^

The International Hip Outcome Tool - 12 (iHOT-12) score was reported by 1 study in 36 patients with a score value of 74.9 ± 1.7.^11^

The rate of return to sport was reported across 289 patients and ranged from 75.6% to 98%. One study that looked at a group of dancers found a correlation coefficient of 0.45 (*P* < 0.01) for years dancing and return to dance rates (Table 3).

**Table 3.** Patient-reported outcomes

Author (Year)	No. of Patient/Hips	VAS Preop	VAS Postop	VAS *P* Value	HOS-ADL Preop	HOS-ADL Postop	∆HOS-ADL	HOS-ADL *P* Value	HOS-SSS Preop	HOS-SSS Postop	∆HOS-SSS	HOS-SSS *P* Value	mHHS Preop	mHHS Postop	∆mHHS	mHHS *P* Value	Return-to-Sport Rates, %
Saks (2022)	47/50 (flexibility sports)	5.3 ± 2.3	1.8 ± 2.1	<0.01	NR	NR	NR	NR	44.5 ± 22.1	78.9 ± 23.0	34.4	<0.01	66.1 ± 13.1	87.8 ± 14.5	21.7	<0.01	75.60
Keating (2020)	25 (22 completed follow-up)	68.8 ± 14.7	21.4 ± 19.9	<0.01	56.5 ± 17.4	86.1 ± 10.2	29.6	<0.01	30.2 ± 20.1	63.8 ± 30.6	33.6	<0.01	57.6 ± 15.1	79.2 ± 12.5	21.6	<0.01	17 patients (77%) overall (35%) higher level (59%) same level (6%) lower level
Mas Martinez (2020)	36 (flexibility sports)	NR	NR	NR	67.5 ± 17.7	89.6 ± 14.7	22.1	0.05	42.3 ± 21.2	75.0 ± 22.6	32.7	0.02	77.1 ± 11.1	93.4 ± 9.9	16.3	0.03	86.1
Larson (2020)	63 dancers (77 hips)	6	2.4	<0.01	72.52 (range, 21-100 points)	91.47 points (range, 53- 100 points)	15.95	< 0.01	49.73 (range, 0-100 points)	79.57 points (range, 25-100 points)	79.57	< 0.01	60.02 (range, 31- 98 points)	85.63 points (range, 47-100 points	25.61	< 0.01	63% same level, 21% lower level level/limited, and 16% unable to return to dance, including 1 retirement
Ukwuani (2019)	64	7.8 ± 6.41	0.9 ± 1.1	0.01	60.5 ± 19.5	92.4 ± 11.8	31.9	<0.01	40.3 ± 20.3	83.5 ± 19.4	43.2	<0.01	57.0 ± 13.6	86.6 ± 13.9	29.6	<0.01	62 patients (97%) returned to dance including 41 of 42 patients (98%) involved in ballet
Frank (2018)	42/45	6.27 ± 2.15	1.40 ± 1.81	<0.01	69.98 ± 18.71	93.84 ± 6.06	23.86	<0.01	48.03 ± 23.69	85.88 ± 12.87	37.85	<0.01	61.75 ± 12.93	89.65 ± 10.89	27.9	<0.01	39 (93%) overall, 19 (45%) higher level, 17 (40%) same level, 3 (7%) lower level. 2 of 3 lost interest 1 of 3 unable due to pain
Weber (2017)	15 (flexibility sports)	NR	NR	NR	NR	NR	26.8 ± 23.6	NR	NR	NR	33.4 ± 30.0	NR	NR	NR	25.8 ± 18.0	NR	14 (93%)
Mohan (2017)	3 (flexibility sports)	NR	NR	NR	NR	NR	NR	NR	NR	NR	NR	NR	NR	NR	NR	NR	NR

HOS-ADL, Hip Outcome Score - Activity of Daily Living; HOS-SSS, Hip Outcome Score - Sport-specific Subscale; mHHS, modified Harris Hip Score; NR, not reported; preop, preoperative; postop, postoperative; VAS, Visual Analog Scale.

### Complications

A total of 4 studies covering 130 patients reported no complications over the course of the study, with follow-up ranging from 12 to 116 months.^3,5,8,13^ One study reported 1 minor complication involving a pudendal nerve injury that resolved by the 6-week follow-up mark.^21^ The remaining 3 studies did not report explicitly on complication rates.^11,15,19^

## Discussion

Patients who participate in flexibility sports demonstrate favorable postoperative patient-reported outcomes after undergoing arthroscopy for FAIS at a minimum of 12 months. In addition, the majority of athletes were able to return to sport at a similar or higher level than presurgery. This supports the hypothesis that hip arthroscopy is beneficial for flexibility sports athletes hoping to return to sport. In addition, the pre- and postoperative differences in the various patient-reported outcomes signify an improvement in important metrics such as pain, quality of life, and function.

Flexibility sports are unique in that they require the use of the hip joint in the extremes of its ROM. Larson et al^8^ reviewed a group of professional dancers and found that more than half (55%) had dysplastic hips and 25% had FAIS. This study also reported that ballet athletes and those with hip dysplasia were the least likely to return to the sport. This may be due to a combination of both bony and soft tissue instability in the setting of the enhanced flexibility requirements of their sport. Similar findings have also been reported in other studies that have found higher incidences of ligamentum teres tears or femoral head cartilage lesions in flexibility sports. This may indicate that these athletes frequently have secondary pathologies that impact their potential recovery and return to preinjury level of sport.^11,12,15,20^ Many factors have been associated with return to sport after FAI surgery, including the duration of participation before injury. Longer participation in a sport and shorter withdrawal before intervention have been shown to be associated with enhanced return to sport.^10,21^

In addition, it is important to consider the difference between professional and amateur athletes. For many professional athletes, there are incentives to return to sport regardless of satisfaction or pain level.^21^ Tjong et al^17^ found 3 main themes for successful return to play: high self-efficacy, adequate social support, and proper expectation management. These themes were seen more commonly in athletes who returned to sport successfully compared with those who had not. These qualitative factors should be considered in addition to the usual quantitative factors reported with return to sport.

This review is consistent with previous evidence regarding the benefits of arthroscopy in the treatment of FAIS. In addition, this review further specifies that arthroscopy shows benefit in the specific subpopulation of flexibility athletes. The strength of this review is that it was a comprehensive analysis of the available literature regarding postoperative outcomes in patients undergoing arthroscopic surgery for FAIS. It employed rigorous methodology and utilized random effects modeling to pool various patient-reported postoperative outcomes. The strict inclusion criteria and pooled data further help describe the efficacy of the treatment, especially in this unique study population.

The main limitations of this review include the relatively low number of studies in the final analysis as well as the significant heterogeneity in the outcomes reported in the studies. Despite this, it represents one of the most robust analyses of this study population. Another limitation is the low levels of evidence included in the review and variability in rehabilitation practices, which limits the comparability of the included studies.

Future directions include larger multicenter prospective studies that focus on scores such as the iHOT and the Hip Disability and Osteoarthritis Outcome Score, which are more specific to the young adult hip. Improvements can also be made with longer term follow-up to assess for potential recurrence of symptoms or any degenerative changes associated with sporting activity. Finally, standardized approaches to surgical treatment and rehabilitation in this population would enhance comparative analyses in the future.

## Conclusion

This review has demonstrated a trend of improvement in patient-reported pain, function, quality of life, and return to sport at a minimum of 12 months among flexibility athletes after hip arthroscopy to treat FAIS.

## Supplemental Material

sj-pdf-1-sph-10.1177_19417381231217503 – Supplemental material for Return-to-Sport Rates AfterHip Arthroscopy for FemoroacetabularImpingement Syndrome in Flexibility Sports Athletes: A Systematic Review Supplemental material, sj-pdf-1-sph-10.1177_19417381231217503 for Return-to-Sport Rates AfterHip Arthroscopy for FemoroacetabularImpingement Syndrome in Flexibility Sports Athletes: A Systematic Review by Muyiwa Ifabiyi, Milin Patel, Dan Cohen, Nicole Simunovic and Olufemi R. Ayeni in Sports Health
