# Supplementary material for: Return-to-Sport Rates After Hip Arthroscopy for Femoroacetabular Impingement Syndrome in Flexibility Sports Athletes: A Systematic Review
Source: Sports Health. 2023 Dec 28;16(6):982–90. doi: 10.1177/19417381231217503 (PMC11531010; doi:10.1177/19417381231217503)
Supplement: sj-pdf-1-sph-10.1177_19417381231217503 – Supplemental material for Return-to-Sport Rates AfterHip Arthroscopy for FemoroacetabularImpingement Syndrome in Flexibility Sports Athletes: A Systematic Review [file sj-pdf-1-sph-10.1177_19417381231217503.pdf]

APPENDIX

Table A1: Search Terms and Results

	Pubmed	Medline	Embase
1) Hip		180165	247218
2) Arthroscopy		33779	42625
3) FAI		3025	4145
4) Femoroacetabular Impingement		3672	4891
5) 3 OR 4		5101	6919
6) 1 AND 2 AND 5	2127	2007	2696
